# Draft Genome of *Rhodococcus rhodochrous* TRN7, Isolated from the Coast of Trindade Island, Brazil

**DOI:** 10.1128/genomeA.01707-15

**Published:** 2016-03-03

**Authors:** Edmo M. Rodrigues, Victor S. Pylro, Priscila T. Dobbler, Filipe Victoria, Luiz F. W. Roesch, Marcos R. Tótola

**Affiliations:** a Departamento de Microbiologia, Laboratório de Biotecnologia e Biodiversidade para o Meio Ambiente, Universidade Federal de Viçosa, Viçosa, Minas Gerais, Brazil; b René Rachou Research Centre, Genomics and Computational Biology Group CPqRR/FIOCRUZ, Belo Horizonte, Minas Gerais, Brazil; c Centro Interdisciplinar de Pesquisas em Biotecnologia, CIP-Biotec, Campus São Gabriel, Universidade Federal do Pampa, São Gabriel, Rio Grande do Sul, Brazil; d Núcleo de Estudos da Vegetação Antártica, Campus São Gabriel, Universidade Federal do Pampa, São Gabriel, Rio Grande do Sul, Brazil

## Abstract

Here, we present a draft genome and annotation of *Rhodococcus rhodochrous* TRN7, isolated from Trindade Island, Brazil, which will provide genetic data to benefit the understanding of its metabolism.

## GENOME ANNOUNCEMENT

The genus *Rhodococcus* is closely related to *Nocardia*, *Corynebacterium*, *Gordonia*, and *Mycobacterium* and belongs to the *Actinobacteria* phylum and *Nocardiaceae* family. *Rhodococcus rhodochrous* is a metabolically diverse species found in different environmental niches, from soil to waste treatment plants (1–3). Such diversity can be used in the bioremediation of hydrocarbons, toluene, and other aromatic compounds. Members of the genus *Rhodococcus* have been shown to synthesize and accumulate triacylglycerol (TAGs) (4), which can be an alternative in the production of biofuels.

The strain *R. rhodochrous* TRN7 was isolated from the coast of Trindade Island, Brazil. It was isolated in a medium containing naphthalene as the sole source of carbon and energy. *R. rhodochrous* TRN7 was able to grow in 13 different hydrocarbons as sole source of carbon and energy (5).

Genome sequencing for *R. rhodochrous* TRN7 was performed using the Ion Torrent PGM platform (Life Technologies). Briefly, the genomic DNA was fragmented by using the Bioruptor UCD-200. The template library was prepared with the Ion Plus fragment library kit and clonally amplified in the One Touch System with the Ion PGM template OT2 400 kit. The amplified library was sequenced using the Ion PGM sequencing 400 kit within the 318 Chip version 2. A total of 5,048,800 reads, ranging from 25 to 484 bp in length, were sequenced. The resulting reads were assembled using the MyPro pipeline software (6). Briefly, sequences were trimmed and filtered, followed by genome assembly. MyPro groups five different genome assemblers: VelvetOptimiser, Edena, Abyss, SOAPdenovo, SPAdes, and SOAP2. Integration of the resulting contigs was performed using CISA and SOAP2. Integration resulted in 173 contigs, totaling 4,871,006 bp, with an average size (*N*_50_) of 70,171 bp, longest contig size of 278,361 bp, and G+C content of 70.2%.

Genome annotation was performed using Prokka (7) and Swiss-Prot (8) and revealed 4 rRNAs, 55 tRNAs, 1 tmRNA, and 5,067 genes, of which 5,007 were protein-coding-sequences, including 509 putative proteins and 1,753 hypothetical proteins. The KEGG Automatic Annotation Server (KASS) (9) was used for pathways analysis, which identified 45 genes related to metabolic pathways. Moreover, 24 genes related to microbial metabolism in diverse environments were found.

KASS also identified the HpaB and BenA-XylX genes as being responsible for the degradation of aromatic compounds, and 17 genes involved in the biosynthesis of antibiotics. Furthermore, several enzymes were identified as being related to biosynthesis and metabolism of fatty acid, such as TAGs, like pyruvate carboxylase, acetyl-CoA C-acetyltransferase, 3-hydroxyacyl-CoA dehydrogenase, and fatty acid synthase. In addition, it also has genes for cytochrome P450 monooxygenase and an Alk gene cluster, which is part of an alkane-degradative system.

Bearing in mind the adaptability of metabolism found in *R. rhodochrous* TRN7, the genome sequencing data in this study will support a better understanding of its metabolism and the application of TRN7 for bioremediation of oil spills, or as a new possible source for the production of biofuels.

### Nucleotide sequence accession numbers.

This whole-genome shotgun project has been deposited at DDBJ/EMBL/GenBank under the accession numbers FBUK01000001 to FBUK01000173. The versions described in this paper are the first versions.
